# Reference Intervals for Conventional Transthoracic Echocardiography and Two-Dimensional Speckle Tracking Echocardiography-Derived Strain Values in the Dutch Sheepdog (‘Schapendoes’)

**DOI:** 10.3390/ani15111524

**Published:** 2025-05-23

**Authors:** Dinand Favier, Celine Brugada-Terradellas, Johannes Vernooij, Alma Hulsman, Giorgia Santarelli

**Affiliations:** 1 Department of Clinical Sciences, Faculty of Veterinary Medicine, Utrecht University, Heidelberglaan 8, 3584 CS Utrecht, The Netherlandsg.santarelli@uu.nl (G.S.); 2 IVC/Evidensia Cardiology Department, Evidensia Dierenziekenhuis Nieuwegein, Nijverheidsweg 1b, 3433 NP Nieuwegein, The Netherlands; 3 Department of Population Health Sciences, Faculty of Veterinary Medicine, Utrecht University, Yalelaan 7, 3584 CL Utrecht, The Netherlands

**Keywords:** breed-specific, M-mode, B-mode, Doppler, TDI, speckle tracking echocardiography, strain, strain rate

## Abstract

Echocardiographic values can vary between dog breeds, making breed-specific reference intervals (RIs) preferable. Two-dimensional speckle tracking echocardiography (2-D STE) is an advanced imaging technique that enables the measurement of myocardial deformation parameters, contributing to the assessment of systolic function. The objective was to determine breed-specific RIs for 2-D, M-mode, and Doppler-derived echocardiographic parameters for Dutch Sheepdogs, and to obtain 2-D STE-derived strain and strain rate values in this breed. Apparently healthy, purebred Dutch Sheepdogs (1–7 years) were recruited. Each dog underwent a physical examination and transthoracic echocardiography. Conventional 2-D, M-mode, and Doppler measurements were obtained; strain analysis was performed with 2-D STE software. RIs were established for conventional echocardiographic parameters; clinically relevant parameters were compared with commonly used RIs. The effects of gender, age, body weight (BW) and heart rate were tested. Sixty dogs were included. Panting and/or tachycardia were observed in 24 dogs, which affected the quality of the analysis to varying degrees (e.g., out-of-sector movement, lung artefacts). The selected parameters for left ventricular (LV) and atrial dimension showed good agreement with published RIs. BW was an independent variable influencing LV dimensions. This study provides RIs for conventional echocardiographic measurements and reports 2-D STE-derived strain and strain rate values obtained in Dutch Sheepdogs. The selected parameters of LV and left atrial dimension showed good agreement with commonly used RIs. Anxious behavior could represent a breed peculiarity to take into account when performing echocardiography, as it can affect image quality.

## Simple Summary

Echocardiography is the most commonly used diagnostic technique for cardiac examination when cardiac disease is suspected. Echocardiographic values in dogs can vary due to differences in body size, thoracic conformation, and activity levels. Therefore, breed-specific echocardiographic reference intervals are preferred. Two-dimensional speckle tracking echocardiography is an advanced imaging technique that allows for the measurement of deformation parameters, contributing to systolic function assessment for the entire ventricle (through global strain and strain rate) or its segments (through segmental strain and strain rate values). The aim of this study was to establish breed-specific reference intervals for conventional echocardiography for the Dutch Sheepdog and compare them with ranges commonly used in canine medicine. Furthermore, two-dimensional speckle tracking-derived strain and strain rate values were obtained. The influence of body weight, heart rate, age, and gender was assessed; inter- and intra-observer variability was determined. Reference intervals were generated from 60 dogs. Body weight was identified as the most significant independent variable for most conventional echocardiographic measurements of cardiac dimensions. Strain analysis was feasible, with the heart rate having notable effects on radial and circumferential strain analyses. Variability was acceptable for clinical use for nearly all conventional echocardiographic measurements, as well as for global strain. However, segmental strain analysis showed greater variability. Panting and tachycardia associated with anxious behavior commonly complicated echocardiographic assessments in Dutch Sheepdogs.

## 1. Introduction

The Dutch Sheepdog is a typical Dutch breed, with a predisposition for patent ductus arteriosus (PDA) [1]. In the authors’ experience, dogs of this breed are also commonly diagnosed with myxomatous mitral valve disease (MMVD). Screening and monitoring of the Dutch Sheepdog breed for these conditions is therefore of great importance.

Echocardiography is one of the most common methods used to screen for the presence of cardiac disease. Quantification of chamber size and the assessment of cardiac function are the cornerstones of cardiac imaging, for which echocardiography is the most commonly used non-invasive modality [2]. Reference intervals (RIs) for 2-D and M-mode are used in clinical practice to establish if cardiac dimensions are within accepted limits of normality; the most commonly used RIs are derived from dogs of diverse canine populations and allometrically scaled based on body weight (BW) [3,4,5]. Canine breed-specific RIs can vary due to differences in body size and thoracic conformation, and the specific purpose of the dog/breed (e.g., athletic dogs). Therefore, caution is warranted when comparing results obtained in a specific dog with RIs obtained in a general dog population [6,7]. Although numerous breed-specific RIs have been published [6,8,9,10,11,12,13,14,15,16,17,18,19,20,21,22,23,24,25,26,27,28,29,30], RIs for the Dutch Sheepdog are currently lacking.

Relevant measurements to be established include the conventional parameters of cardiac dimension and function. Two-dimensional (2-D) speckle tracking echocardiography (STE), a relatively recent advanced echocardiographic technique, is gaining popularity within companion animal cardiology and is increasingly used to assess both left and right ventricular myocardial function. With 2-D STE, speckles (‘fingerprints’) are traced throughout the cardiac cycle, providing information about myocardial lengthening and shortening through parameters such as strain and strain rate (SR). Cardiac magnetic resonance imaging (MRI) tagging is regarded as the gold standard for the non-invasive assessment of systolic deformation in humans [31]. However, it has been shown that 2-D STE can also provide accurate and angle-independent measurements of regional myocardial strain [31]. Furthermore, cardiac MRI requires prolonged general anesthesia in dogs, demands highly trained operators, and entails higher costs for owners.

Strain describes the deformation of an object normalized to its original length, while SR describes the rate at which deformation occurs, i.e., how quickly the deformation takes place. Strain is a dimensionless entity, reported as a fraction or percentage [32]. Myocardial strain is defined as the relative change in length of a myocardial segment and can be measured via 2-D STE for specific cardiac regions (segmental strain), or across the entire ventricle (global strain); furthermore, various layers can be investigated, including the myocardium and the endocardium [32]. Deformation can be calculated in the longitudinal, circumferential, and radial directions [33]. In certain dog breeds, 2-D STE-derived strain and SR have been investigated [34,35,36,37,38,39,40]. Global longitudinal strain (GLS) has been shown to be a feasible and reproducible parameter to evaluate systolic function in healthy Doberman Pinschers [34]. It is important to note that the results of strain analysis can vary depending on the imaging system and software used, with significant differences existing between vendor-dependent and vendor-independent software programs [41,42].

The objective of this study was to obtain conventional 2-D, M-mode, spectral and tissue Doppler-derived echocardiographic measurements, as well as vendor-independent 2-D STE-derived global and segmental longitudinal, circumferential, and radial strain and SR values in Dutch Sheepdogs, and to establish breed-specific RIs for conventional measurements. A set of 2-D and M-mode-derived parameters were also compared with commonly used RIs obtained from dogs of various breeds. Furthermore, the influence of BW, heart rate (HR), age, and gender on the echocardiographic parameters investigated was tested and inter-observer and intra-observer variability was determined.

## 2. Materials and Methods

### 2.1. Animals

Over a period of 1 year, 60 apparently healthy purebred Dutch Sheepdogs, both male and female, were enrolled in a prospective observational study. Although a sample size of at least 120 dogs is recommended to establish RIs with a 90% confidence interval (CI) using a nonparametric method, this number of dogs was deemed difficult to achieve for this relatively small Dutch dog breed. Therefore, a sample size of 60 dogs was considered both feasible to recruit in agreement with the breeding association and sufficient considering the American Society for Veterinary Clinical Pathology (ASVCP) guidelines, when alternative methods are used to determine 90% CI. It is worth noting that a minimum of 39 samples is required to determine a 95% nonparametric RI [43].

All dogs were recruited through the association ‘the Dutch Schapendoes’ and examined at the Cardiology Service of the Clinical Sciences Department of the Faculty of Veterinary Medicine, Utrecht University. All owners signed an informed consent and institutional ethical approval was obtained (WP 10813-2023-02).

### 2.2. Inclusion and Exlusion Criteria

All included Dutch Sheepdogs were required to be free of known cardiac or extra-cardiac diseases. They were also needed to be older than 1 year (fully grown) and under 7 years of age (to decrease the possibility of other, asymptomatic disorders and impaired diastolic function). Additionally, dogs could not be receiving any medication, except for antiparasitic treatment or vaccinations, and female dogs could not to be pregnant.

### 2.3. Examination

Physical examination, including cardiac auscultation, and echocardiography with simultaneous one-lead electrocardiogram (ECG) recording were performed on all Dutch Sheepdogs. For the echocardiographic examination, a GE Logiq S8 ultrasound machine equipped with 1.5–4.5 mHz and 2.4–8.0 mHz phased array transducers was used (GE Healthcare). The examinations were performed by a board-certified cardiologist (GS), or by a cardiology resident (DF) or intern (CB) under direct supervision of a board-certified cardiologist (GS, AH).

Echocardiographic examinations were performed with dogs in left and right lateral recumbency on a table with a cut-out for scanning from beneath. All dogs were positioned and restrained without sedation. The standard recommended transthoracic echocardiographic views (right parasternal, subcostal, left apical parasternal and left cranial parasternal) were performed according to the recommendations for standards in transthoracic 2-D echocardiography in dogs [44]. In cases of visible regurgitations, the valve anatomy and leaflet motions were closely inspected with 2-D from multiple views, and regurgitations were assessed semi-quantitatively with color Doppler by measuring the regurgitant jet size. In cases of atrioventricular insufficiency, regurgitation was classified as trivial if the jet did not extend more than 1 cm past the annulus; mild if it occupied <20% of the atrium, moderate if 20–40%; and severe if >50% [14,45]. For aortic or pulmonic insufficiencies, the assessment was similar but based on the relationship of the jet size with the size of the outflow tract or ventricle instead of the atrium [45,46]. Dogs classified as having trivial mitral and/or aortic valve regurgitations and trivial-to-mild pulmonary and/or tricuspid valve regurgitations were allowed in the study, provided their valves appeared structurally normal.

The internal short-axis diameter of the left atrium (LA) and aortic root (Ao) were measured in one frame in a right parasternal short-axis view (SAX) on the first frame after aortic valve closure. The LA/Ao ratio was calculated [47]. The maximal left atrial anteroposterior diameter (LAD) was measured in a right parasternal long-axis four-chamber (PLAX) view at end-systole (1–2 frames before mitral valve (MV) opening) at the widest dimension, parallel to the MV annulus from the inner wall (endocardial border) of the interatrial septum to the inner wall of the posterior free wall. The distance from the blood–tissue interface to the blood–tissue interface was used [47,48,49]. Left ventricular (LV) volumes at end-systole (ESV, corresponding to last frame before MV opening) and at end-diastole (EDV, corresponding to the onset of QRS or the time of MV closure) were measured in both PLAX and left apical four-chamber (A4C) views by applying the modified Simpson’s method of discs [50]. M-mode measurements of the interventricular septum (IVS), left ventricular internal dimension (LVID), and left ventricular free wall (LVFW) in diastole (d) and systole (s) were performed at the chordae tendineae level using the leading edge—leading edge method; E-point to Septal Separation (EPSS) was measured at the level of the MV in a SAX view [4,51,52]. LVID normalized for BW in d and s according to Cornell et al. [3] were recorded (LVIDdN and LVIDsN). HR was recorded as the R-R interval on the 1-lead simultaneous ECG, which was obtained from the LV M-mode trace. The tricuspid annular plane systolic excursion (TAPSE) was determined by an M-mode recording of the lateral aspect of the tricuspid annulus in the A4C view centered on the right ventricle (RV) [53].

To obtain Doppler measurements, pulsed wave (PW) Doppler was applied at the level of the pulmonary valve (PV) in the SAX view, continuous wave (CW) Doppler over the aortic valve in the subcostal view, and PW Doppler at the level of the aortic valve in the left apical five-chamber (A5C) view. PW Doppler was also used to record transmitral inflow at the tips of the mitral leaflets in the A4C view. Mitral inflow measurements, including peak early (E) and peak late (A) diastolic velocities were obtained when possible; the E/A ratio was calculated. The same procedure was followed at the level of the tricuspid valve to obtain the tricuspid inflow velocities. Isovolumic relaxation time (IVRT) was measured as the time period from the Doppler signal of aortic valve closure to the beginning of the transmitral E wave on images obtained with the PW sample volume placed between the LV inflow and outflow tracts [54].

PW-tissue Doppler imaging (TDI) was performed from A4C views, with the PW gate placed at the basal interventricular septum (IVS), the LV free wall (LVFW), and the RV free wall (RVFW). PW-TDI recordings were excluded from analysis if they had summation of early (E’) and late diastolic (A’) wave signals [55,56].

The mean of three measurements was calculated and recorded for each B-mode, M-mode, Doppler, and TDI value.

**Figure 1:**
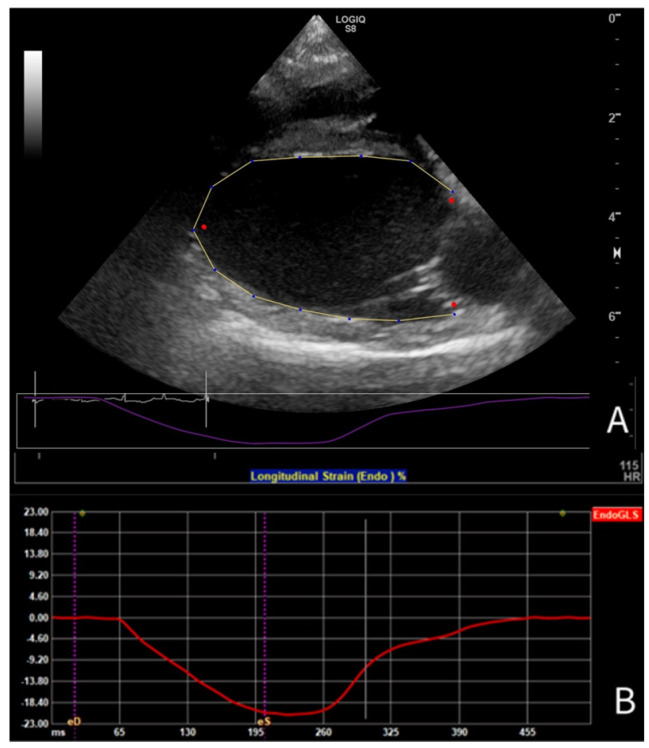
(**A**) Tracing of the LV endocardial border in PLAX view for longitudinal strain analysis using 2-D speckle tracking echocardiography in a Dutch Sheepdog, (**B**) with the corresponding endocardial global longitudinal strain curve during one cardiac cycle.

**Figure 2:**
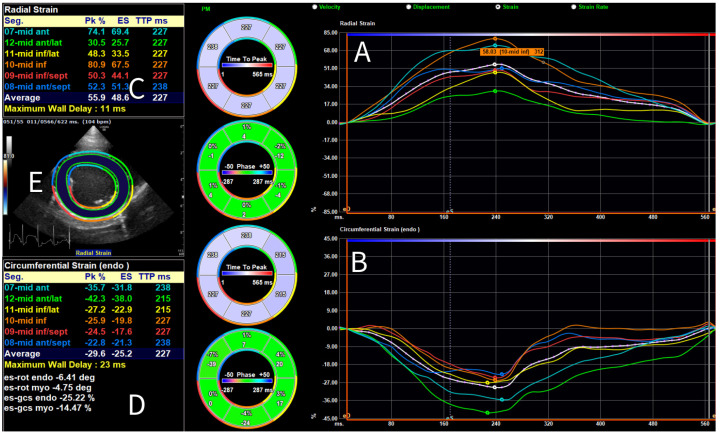
Global and segmental radial and circumferential strain analysis performed using 2-D speckle tracking echocardiography for a Dutch Sheepdog. Myocardial radial (**A**); and endocardial circumferential (**B**) strain curves are displayed for the average (white) and individual segments (color-coded), shown over one cardiac cycle. Dots on each curve indicate the timing of the peak strain of the corresponding segment. On the left, values for peak strain (Pk) and end-systolic strain (ES) are shown for both the average and the individual segments for radial (**C**); and circumferential strain (**D**). On the left, in the center, a short-axis view of the left ventricle is shown (**E**) with a superimposed region of interest and corresponding color mapping of the segments.

For 2-D STE-derived strain analysis, multibeat loops were obtained from SAX, PLAX, and A4C views, and the A4C view focused on the RV, with a frame rate (FR) of at least 50 frames per second. Analysis was conducted using vendor- independent 2-D software (2D Cardiac Performance Analysis 2.51, TomTecArena^TM^ 2022, TomTec Imaging Systems GmbH, Unterschleissheim, Germany). The loops were analyzed by a board-certified cardiologist trained in 2-D STE (GS), as follows. One cardiac cycle was manually selected, and end-systolic and end-diastolic frames were identified. End-diastole was defined using the peak of the QRS complex on simultaneous ECG [32], combined with a subjective assessment of the largest LV dimension; end-systole was determined using the end of the T-wave and the smallest luminal dimension. The analysis software was used to include the endocardial border of the LV for longitudinal strain analysis in the PLAX (Figure 1) and A4C views, for circumferential strain analysis in the SAX view, and of the RV for longitudinal strain analysis in the A4C view focused on the RV. For radial strain analysis of the LV in the SAX view, the entire myocardium was included (Figure 2). For most analyses, default software settings were used; for longitudinal analysis from the PLAX view, a method previously described for use in dogs [57] was employed and the corresponding segments were renamed accordingly. Semi-automatic contour drawing was used for all views, with three landmarks placed. The software generated a proposed endocardial or myocardial contour. These proposed borders were reviewed and adjusted by the investigator if needed. The software divided the ventricles into six segments and provided strain and SR values for each segment (defined as the average value of strains and SRs in that segment [32]), along with the endocardial and myocardial strain and SR for the entire ventricle (calculated both as the average of the segmental values and as the value obtained from the entire ventricle as a unique segment). The operator ensured that the region of interest accurately followed the myocardial movements throughout the cardiac cycle. Manual correction of time intervals and endocardial borders in end-systole and end-diastole was performed if necessary.

Peak strain values, defined as the highest value of the strain curve during one cardiac cycle [32] were chosen by the authors over peak systolic or end-systolic strain values. Furthermore, to express global strain and SR, the average value of the segmental strains and SRs provided by the software was chosen. Accordingly, peak segmental strain values, peak global systolic strain, and SR were recorded. All measurements were obtained three times; the mean value was used for the analysis.

### 2.4. Measurement Variability

Inter- and intra-observer measurement variability for conventional M-mode, B-mode, Doppler and TDI measurements was determined from 6 arbitrarily selected echocardiograms. Each echocardiogram was subjected to two repeated analyses by a single investigator (DF) for intra-observer variability: on the same day (within-day variability) and on 2 separate days at least one month apart (between-day variability). Additionally, the echocardiograms were analyzed by a second investigator (CB) for inter-observer variability. Both investigators were blinded to previous measurements. For strain parameters, variability was calculated from 6 other stored and arbitrarily selected echocardiograms. Each echocardiogram was subjected to two repeated analyses by an experienced investigator (GS) on the same day (within-day variability) and on two separate days at least one month apart (between-day variability). Additionally, the echocardiograms were analyzed by a second investigator trained in 2-D STE-derived strain analysis (DF) to assess inter-observer variability. The observers were blinded to the results of the previous measurements when performing the analysis; however, the same cineloops were used each time.

### 2.5. Statistical Analysis

Statistical analyses were performed using commercial statistical software (IBM SPSS Statistics Version 28.0.1.0). Normal distribution for all variables was tested using the Shapiro–Wilk normality test (if *p* < 0.05 normal distribution was not present), with outliers identified using Tukey’s method. Descriptive statistics were generated; the median, upper and lower reference limits, as well as a 90% CI for limits, were calculated using an open-source application for Excel (Reference Value Advisor version 2.1) [58]. The nonparametric method was applied to determine RIs, with a bootstrap method for determining the CI of the limits of the nonparametric RIs. Data are presented as summary statistics (median, standard deviation, interquartile range (IQR), minimum–maximum (min–max)). When a normal distribution was present, mean and RIs were established as two double-sided 95% RIs with a 90% CI of the lower and upper limits based on the non-parametric percentile method following the reference interval guidelines of the ASVCP [43]. If a normal distribution was not present, data were presented as median, along with the IQR and min–max values without referencing RIs. Given that strain analysis results can vary depending on the imaging system and software used, and because of a relatively low number of reliable analyses in this study, these parameters were also reported as median, IQR, and min–max values instead of RIs.

The effects of gender, age, BW and HR on echocardiographic variables were evaluated using simple linear regression. Because of the high number of parameters tested, a Bonferroni correction was performed to reduce the probability of a type I error. An independent samples Student’s *t*-test was carried out to compare the mean between the male and female population (*p* < 0.05) for M-mode and B-mode measurements. Multiple linear regression was also performed for M-mode and B-mode measurements.

A set of M-mode and B-mode echocardiographic variables was chosen by the authors as they were deemed especially useful for the assessment of dogs with PDA and MMVD, including LA/Ao, LAD, EDV, ESV, and LVIDd normalized for BW. These parameters were plotted with graphs to compare the values obtained for the Dutch Sheepdog with previously published RIs from diverse canine populations [3,4,5,49,59] and with the limits used for the staging of MMVD (LA/Ao < 1.6, LVIDd normalized for BW < 1.7) [3,60,61].

Mean values obtained by GLS performed in A4C and PLAX views were compared using a paired Student’s *t*-test (*p* < 0.05).

To assess variability, mean and standard deviation (SD) values of the repeated examinations for conventional and 2-D STE-derived measurements were used to determine inter-observer, within-day, and between-day intra-observer coefficients of variation (CVs). The CVs were calculated as follows: CV = (SD of the measurements/average of measurements) × 100% [57]. Mean CV values <15% were considered adequate for clinical use, with CV < 5% (very low variability); 5–15% (low variability); 15–25% (moderate variability); and >25% (high variability) [62,63].

## 3. Results

A total of 62 Dutch Sheepdogs were initially recruited. One dog was subsequently excluded due to the presence of a murmur on cardiac auscultation (caused by a left-to-right shunting PDA, as confirmed by echocardiography) and one dog was excluded because of pregnancy. The remaining 60 dogs were included in the study (35 females [13 neutered] and 25 males [3 neutered]). The median age was 37.5 months (range 14–81 months), the median BW was 16.4 kg (range 9.7–23.9 kg), and the median HR was 129 beats per minute (bpm) (range 71–240 bpm).

In 24/60 dogs, the quality of the echocardiographic examination was somehow compromised by anxious behavior leading to intermittent panting, frequent movement, and tachycardia, resulting in the later exclusion of some measurements. Fourteen dogs had visible valve regurgitations that did not compromise inclusion according to the criteria explained in the Materials and Methods section. Such regurgitations were observed at the tricuspid valve (4/60, 6.7%), mitral valve (7/60, 11.7%) and pulmonary valve (7/60, 11.7%). Three dogs (3/60, 5 %) had a maximum velocity over the aortic valve with the CW Doppler slightly above the commonly used reference value of 2.0 m/s (2.10, 2.27, 2.39 m/s, respectively) [64]), without visible structural abnormalities of the LV outflow tract and/or aortic valves, and without insufficiency.

### 3.1. Reference Intervals for Conventional Echocardiographic Parameters

M-mode, B-mode and conventional Doppler variables were obtained from 60 dogs. However, certain values were excluded from analysis when the image quality was insufficient and the measurement deemed unreliable (see Tables for total number of dogs for each parameter). Due to tachycardia, mitral and tricuspid inflow velocities were often unavailable due to the fusion of E/A waves. Most variables showed a normal distribution, except for EPSS, FS, HR, EDV and ESV in the PLAX view, and tricuspid A wave velocity. Additionally, TDI measurements were obtained; however, the measurements were sometimes considered unreliable and therefore excluded due to poor image quality, weak signals and/or fusion of the E’ and A’ waves. Multiple variables did not show a normal distribution. Table 1 summarizes the M-mode and B-mode echocardiographic variables, while Table 2 presents the results of the conventional (PW/CW) Doppler and TDI Doppler analysis.

### 3.2. Results of Strain Analysis

Strain analysis was successfully performed in 56 out of 60 dogs. The first three echocardiograms could not be analyzed with the available software due to faulty export settings, later adjusted. Furthermore, in one additional dog, all the images were of insufficient quality due to severe panting and tachycardia. Strain analysis could be reliably performed in all directions in 32 dogs, while in 24 dogs, analysis was incomplete in varying degrees due to factors such as poor image quality, extreme panting, out of sector movement and/or inadequate one-lead ECG recording. Multiple variables did not show a normal distribution. Table 3 summarizes the global strain and global SR values/parameters for the different directions and views analyzed. Results from the segmental analysis are available in the Appendix A Table A3.

### 3.3. Effects of Body Weight, Age and Heart Rate

Significant associations were observed between multiple echocardiographic variables and BW and HR (Table 4 presents the significant parameters after a Bonferroni correction) in the univariate analysis. BW showed effects for several M-mode and B-mode measurements describing LV and LA dimensions and diameters, with increasing BW being associated with larger cardiac dimensions. HR showed modest but significant effects on several M-mode and Doppler measurements, with a higher HR associated with lower LVIDs and LVIDd, as well as slight increases in FS and a few Doppler-derived parameters (see Table 4). Gender and age did not show significant associations after the Bonferroni correction was performed. BW, age, and gender revealed no significant effects on strain parameters. However, HR had small effects on some radial and circumferential strain values, with a higher HR correlating with lower circumferential strain values and higher global radial SR (see Table 4). 

An independent Student’s t-test comparing BW, multiple M-mode measurements (IVSd, LVIDd, LVPWd, IVSs, TAPSE), B-mode dimensions and volumes (Ao, ESV, EDV), and TDI-derived E′ of the IVS identified significant differences between males and females, with male dogs showing higher values compared to female dogs. Conversely, the calculated parameter LVIDdN (M-mode), normalized for BW according to allometric scaling [3], showed no significant differences between genders. 

Multiple linear regression for conventional measurements confirmed BW as the most significant and important independent variable for most measurements for LV and LA diameters and volumes. Gender was not a significant variable with multiple linear regression.

### 3.4. Comparison with Previously Published Reference Intervals

**Figure 3:**
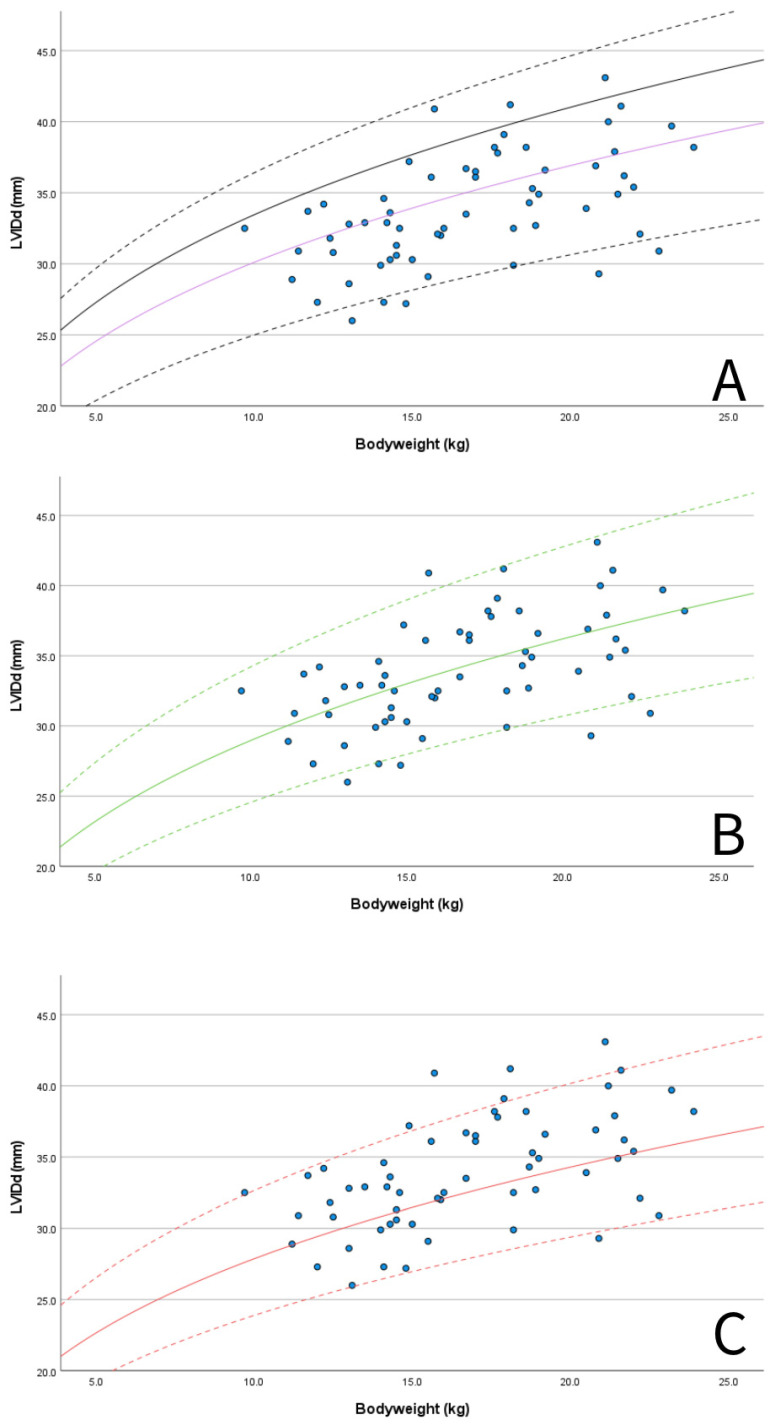
Scatter plots showing M-mode-derived left ventricular internal dimension in diastole (LVIDd) for BW (blue dots): (**A**) 50% line (purple solid line) and 95% prediction intervals according to published RIs using allometric scaling methods in multiple breeds by Cornell et al., 2004 (purple solid line 50% LVIDdN 1.53, dashed black lines 95% RI LVIDdN 1.27–1.85, scaling exponent 0.294) [3] and also the clinically used upper limit for left ventricular enlargement in MMVD (solid black line, LVIDdN 1.7 according to Cornell et al., 2004) [3,61]; (**B**) 50% line (green solid line) and 95% prediction intervals according published RIs using allometric scaling methods in multiple breeds by Esser et al., 2020 (green solid line 50%, LVIDdN 1.38, dashed green lines 95% RI LVIDdN 1.17–1.63, scaling exponent 0.322) [4]; and (**C**) 50% line (red solid line) and 95% prediction intervals according published RIs using allometric scaling methods in multiple breeds by Visser et al., 2019 (red solid line 50%, LVIDdN 1.40, dashed red lines 95% RI LVIDdN 1.20–1.64, scaling exponent 0.299) [5].

**Figure 4:**
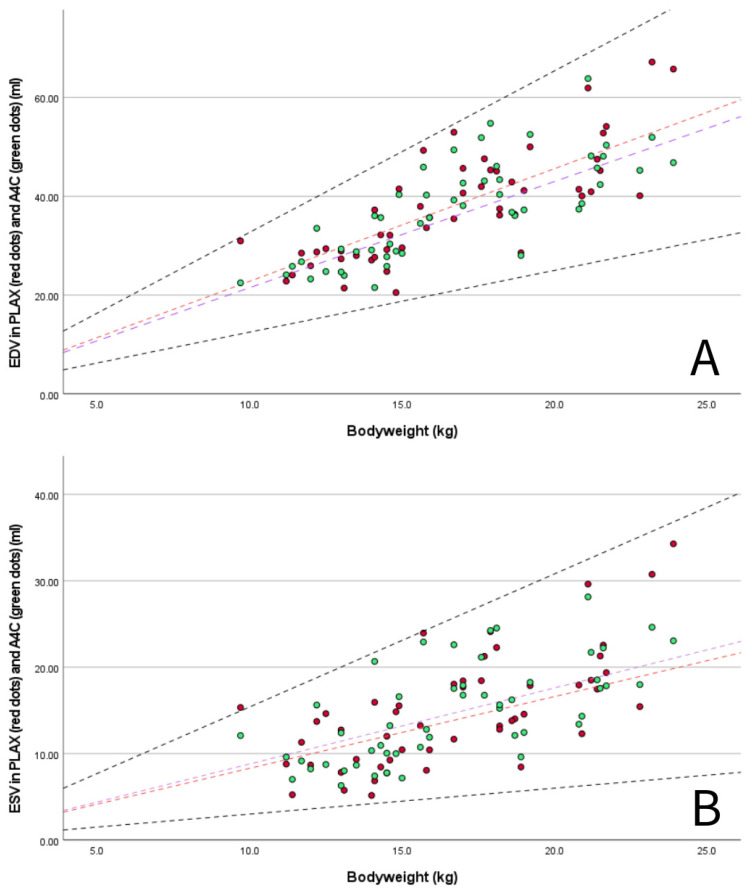
(**A**) scatter plot showing measured end-diastolic volumes (EDV) of the LV in PLAX (red dots) and A4C view (green dots) for BW with published 95% prediction intervals for PLAX view (dashed black lines, 95% RI EDV/kg 1.25–3.27) in multiple breed populations (1211 non-sighthound dogs) using normalization for BW according Wess et al., 2021 [59]. Lines represent the median EDV/kg in Dutch Sheepdogs for PLAX (red dashed line, 2.28 mL/kg) and published median in multiple breed populations by Wess et al., 2021 [59] (purple dashed line, 2.15 mL/kg); and (**B**) scatter plot showing measured end-systolic volumes (ESV) of the LV in PLAX (red dots) and A4C view (green dots) for BW with published 95% prediction intervals for PLAX view (dashed black lines, 95% RI ESV/kg 0.30–1.54) in multiple breed populations (1211 non-sighthound dogs) using normalization for BW according Wess et al., 2021 [59]. Lines represent the median ESV/kg in Dutch Sheepdogs for PLAX (red dashed line, 0.87 mL/kg) and published median in multiple breed populations by Wess et al., 2021 [59] (purple dashed line, 0.88 mL/kg).

**Figure 5:**
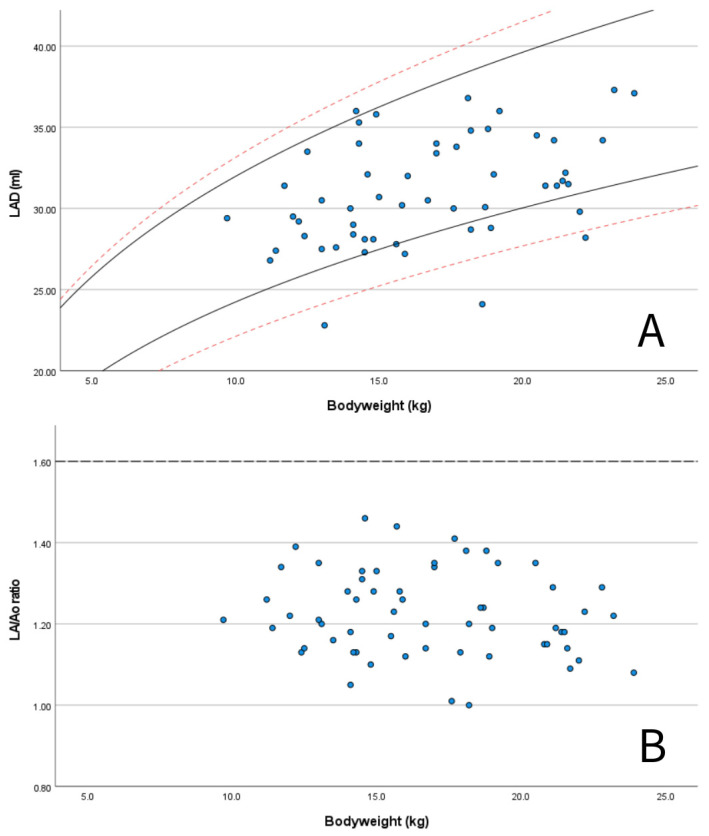
Scatter plots showing: (**A**) measured values of left atrial anteroposterior diameter (LAD) for BW for the Dutch Sheepdogs in this study (blue dots) with published 95% prediction intervals for normalized scaling methods in 122 healthy dogs of different breeds (solid black lines according Visser et al., 2019 [5]) and in 330 healthy dogs of different breeds (red dashed lines, according to Marchesotti et al., 2019 [46]); and (**B**) measured values of LA/Ao ratio for BW for the Dutch Sheepdogs in this study (blue dots) with the generally used upper reference for left atrial enlargement according to the ACVIM consensus guidelines for myxomatous mitral valve disease (dashed line) [51].

M-mode measurements of LVIDd showed good agreement when plotted in graphs against published RIs normalized for BW obtained in diverse canine populations by Cornell et al. (scaling exponent 0.294) [3], Esser et al. (scaling exponent 0.322) [4] and Visser et al. (scaling exponent 0.299) [5] (Figure 3A–C). The mean value of 1.49 for LVIDdN obtained in Dutch Sheepdogs in this study is slightly lower than the mean value of 1.53 obtained by Cornell et al. in a diverse population of dogs, showing statistical significance on a one-sample t-test. EDV in PLAX and A4C views was within the RIs based on normalization for BW proposed by Wess et al. (Figure 4) [59]. LAD normalized for BW also showed good agreement with previously published RIs in 330 healthy dogs [49] and 122 healthy dogs [5], both from various breeds (Figure 5A).

LA/Ao ratios in Dutch Sheepdogs were always below the commonly used limit of normality of 1.6 (Figure 5B) [60,61]. Additionally, 57 out of 60 dogs (95%) had a LVIDdN value below the limit of 1.7 [3]) used as a criterion for LV chamber enlargement as recommended by the ACVIM consensus guidelines for the staging of MMVD (Figure 3A) [60,61].

### 3.5. Comparison of GLS Obtained from A4C and PLAX Views

In Dutch Sheepdogs, GLS strain values from the PLAX view were higher than those obtained from the A4C view. A paired Student’s *t*-test confirmed a statistically significant difference between the mean GLS obtained from the two views (*p* < 0.001).

### 3.6. Intra- and Inter-Observer Variability

Intra-observer variability (within day and between day) was very low (<5%) or low (5–15%) for conventional echocardiographic measurements. Inter-observer variability was also very low or low for B-mode, M-mode, and Doppler-derived measurements except for EDV and ESV from the A4C view, IVRT, and MV E/A ratio (>15%) (see Appendix B Table A4).

The intra-observer variability (within-day and between-day) and inter-observer variability for the strain analysis was very low or low for global strain and SR values in all views (see Appendix B Table A5). Intra-observer and inter-observer variability for the different segments was higher but remained below 15% for most parameters. However, for the radial and longitudinal segmental analysis of the LV from A4C views, and RV longitudinal strain analysis, multiple segments showed moderate variability (15–25%, inter- and intra-observer).

## 4. Discussion

This study provides RIs for conventional 2-D, M-mode, spectral Doppler and TDI-derived echocardiographic parameters for Dutch Sheepdogs. Furthermore, segmental and global strain and SR values obtained by 2-D STE are reported. The results of this study will help clinicians in the assessment of both congenital and acquired heart disease in dogs of this breed.

It is important to highlight that a significant number of dogs showed anxious behavior that made it difficult in some cases to obtain 2-D images and Doppler profiles of a high enough quality to guarantee reliable measurement. High heart rates, panting, and frequent movement were often observed; such behavior could represent a breed peculiarity to take into account when performing echocardiography in these dogs. Despite these challenges, it was possible to obtain most conventional measurements, which allowed for the calculation of breed-specific RIs. However, strain analysis was often incomplete.

Good image quality is extremely important for reliable strain analysis [65]. The high-stress behavior often hindered the attainment of high-quality loops of the beating heart without out-of-sector movement of the myocardium and/or lung artefacts. Additionally, there was a learning curve in recording, processing, and transferring the video loops for offline processing, leading to the exclusion of the first three dogs. Because of the reduced number of dogs from which reliable strain analysis was possible from all directions, and for reasons explained in the following paragraph, we decided not to calculate RIs for these parameters, but to report them as mean, median with IQR, and minimum–maximum.

Two-dimensional STE is increasingly being used to asses myocardial function in dogs; there are currently only a few studies reporting breed-specific values for strain analysis in dogs [34,37]. One study in Doberman Pinschers recently demonstrated that 2-D STE is a feasible and reproducible technique to assess systolic function in this breed [34]. It is important to note that published values for 2-D STE cannot easily be compared across studies. First, small but statistically significant variations among vendor-dependent software platforms and versions should be considered, as well as differences between vendor-dependent and vendor-independent systems [41,42]. Additionally, various parameters can be obtained with strain analysis; the specific measurement chosen should be clearly reported. For example, strain could be expressed as end-systolic strain, which refers to the strain value measured at end-systole; peak systolic strain, which corresponds to the highest value during systole; and peak strain, which corresponds to the highest value of strain throughout the entire cardiac cycle [32]. Peak strain values are equal to, or higher than, end-systolic strain values. It is worth mentioning that end-systolic strain is the recommended parameter by the Task Force to standardize deformation imaging in humans [32]. However, in our study, peak strain was used, as this is more commonly reported in veterinary studies [41,57,65] and some software programs present peak values by default [34].

For clarity, the authors want to emphasize that this study reports segmental and global longitudinal, circumferential, and radial peak strain, as well as SR values, obtained using TomTec 2-D CPA 2.51 (TomTec’s CardioArena^TM^, 2022).

The mean GLS and GCS obtained in this study were higher (more negative) than those reported in previously published studies assessing healthy dogs [35,36,37,39,40], although no statistical analysis was performed to demonstrate this because of differences in the software used and the type of parameters chosen to express strain. However, the GLS values observed in this study seem comparable to those reported by Hertzsch et al. (2022) in Doberman Pinschers [34] and Santarelli et al. (2018) in dogs of various breeds [57]. Interestingly, both studies used the same vendor-independent platform, although not always the same version of it. GRS values reported in different breeds across several studies [35,37,38,39,40,66,67,68] showed broad ranges, with the values obtained for Dutch Sheepdogs falling within this spectrum.

In this study, the authors decided to also perform longitudinal strain analysis in the PLAX view, which is not routinely used for this purpose. Strain analysis from this view is not performed in humans and has not been pre-programmed in the TomTec software. However, LV longitudinal strain analysis from PLAX has been described as a reliable technique for horses [69], goats [70], and dogs [57] using the default settings for longitudinal analysis from four-chamber views. The use of this view offers an alternative when images obtained from the A4C view are not of high enough quality due to lung artefacts [57]. In this study, inclusion of this view allowed for longitudinal analysis in 13 dogs when the A4C view was not assessable. However, in accordance with the results previously published, the deformation parameters in A4C and PLAX view were significantly different in our study and are therefore not interchangeable [57].

Concerning conventional echocardiography, trivial to mild regurgitations were seen in 14/60 (23%) dogs on different valves. These regurgitations were considered hemodynamically insignificant, most probably physiological, and were in line with findings in other studies [10,29]. Due to panting and artefacts on the color Doppler examination, it can be assumed that several trivial to mild regurgitations were missed and that the prevalence of these physiological regurgitations might be higher. However, it is important to note that serial examinations over time could be the only way to differentiate whether a minor (mitral) regurgitation will not be progressive and will not become significant in the future [71]. Furthermore, in 3/60 (5%) dogs, the velocity over the aortic valves was slightly above the commonly used reference value of 2.0 m/s. However, this was also considered hemodynamically insignificant and could have been influenced by the stressed behavior observed in these dogs, as has also been described in Boxer dogs [72].

Given that the evaluation of LV dimensions is of the outmost importance when evaluating diseases such as PDA and MMVD, commonly used parameters obtained using M-mode and B-mode-derived parameters (LVID, EDV and ESV) were plotted against BW and compared with published 95% prediction intervals obtained in diverse canine population studies [3,4,5,59]. Only one dog exceeded the upper 95% RI according to Esser et al., three dogs exceeded the 95% RI and three dogs were at the upper end of the 95% RI according to Visser et al., while all dogs remained below the upper 95% RI for the prediction interval reported by Cornell et al. However, if we had been screening for the presence of LV enlargement in the presence of MMVD, 3 out of 60 dogs (5%) in our study would have been diagnosed with LV enlargement according to the ACVIM consensus guidelines [61]. Therefore, using the EPIC criteria in Dutch Sheepdogs could lead to misclassification and influence treatment decisions [60,61]; the use of these breed-specific RIs should be preferred.

Furthermore, in this study, the LA/Ao ratio was consistently below the widely accepted normal limit of <1.6 [47], with a 95% RI of 1.01–1.45. Therefore, when using an upper reference limit of 1.6, it is possible that Dutch Sheepdogs with mild atrial enlargement may be missed. In these cases, the use of breed-specific RIs provided in this study could also be of clinical value. Another B-mode-derived measurement used to assess LA dimension is LAD, which can aid in determining whether LA enlargement is present in dogs. The LAD measurements obtained in this study, plotted against BW, often fell within prediction intervals previously reported [47], with only one dog having a value exceeding the upper 95% prediction interval of one of these studies. While in most of the dogs, the measurements for LV and LA dimension fell within the widely used accepted limits of normality for dogs of any breed, the breed-specific RIs for LAD published in this study can help to prevent misclassifications.

The effects of gender, age, BW and HR on selected M-mode and B-mode-derived measurements were examined. The results showed that BW was a powerful independent variable with significant effects on different M-mode and B-mode measurements. Additionally, HR also showed some significant effects. These findings are consistent with previous studies conducted on single breeds [16,18,29,30] and on diverse canine breeds [3,4,5,59]. Although the mean measurements of LV and LA dimensions in both B-mode and M-mode were lower in female dogs compared to male dogs, this was due to the difference in BW between the two groups in the study.

HR also showed significant effects on circumferential and radial strain values, as previously observed [66]. Although age is reported as a factor affecting strain analysis in humans [73,74], this could not be demonstrated in this study on Dutch Sheepdogs, which is consistent with findings for other dog breeds [34,39]. However, the exclusion of dogs younger than one year and older than eight years has most likely affected the results of this study.

The intra- and interobserver CVs obtained in this study for most conventional echocardiographic measurements were appropriate for clinical use and were better than, or aligned with, previously reported CVs [18,75,76]. Only minimal B-mode and Doppler-derived measurements showed CVs above the selected limit of 15% [62]. The higher variability of ESV and EDV obtained from A4C in this study compared with that reported in a previous study [59] could be explained by the anxious behavior in a high proportion of the dogs, leading to less precise measurements in images of reduced quality.

Intra-observer and interobserver variability also appeared to be acceptable for clinical use for nearly all global strain and SR parameters, consistent with previous studies [34,41,57,68,77,78,79]. However, repeatability and reproducibility were less acceptable for RV SR and a high proportion of segmental strain values, especially for radial strain analysis. Based on these findings, 2-D STE would seem to be less applicable for the assessment of cardiac segmental function in Dutch Sheepdogs, which is also consistent with previous reports for dogs [57].

This study has limitations. First, the study was conducted at a single center and the sample size was relatively small, as has already been mentioned in the Materials and Methods section. In fact, at least 120 dogs are preferred to create 95% RIs based on the guidelines of the ASVCP [43]. However, the dogs were recruited from all over the Netherlands, and it would have been challenging to recruit more than 60 dogs from this relatively small Dutch dog breed. RIs can also be created with alternative methods while respecting the guidelines of the ASVCP. Therefore, we still believe that the obtained RIs provide a valuable tool for cardiac evaluation in this relatively small breed. Furthermore, although only dogs without clinical complaints and/or abnormalities during the physical examination and echocardiography were included, there is still a possibility that dogs with incipient or subclinical diseases were included. An additional limitation is that for the strain analysis, we used a proposed FR of > 50 frames per second. FR is a crucial factor in obtaining echocardiographic images for strain analysis, as 2-D STE relies on tracking changes in the speckle pattern position between frames. In our study, multiple dogs had an increased HR; however, we did not adjust the frame rate in the absence of clear guidelines on how to do so properly. It is known that studies involving an increased HR require a proportional increase in FR [32]. Lower FR may lead to unsatisfactory 2-D STE due to reduced temporal resolution and possible speckle drop out, resulting in an underestimation of strain values. Conversely, a too-high FR (>100 frames per second) may also cause problems for identifying the speckle pattern [80]. Therefore, it is important to maximize image quality and other settings during echocardiography whilst subjectively monitoring myocardial tracking [80]. Furthermore, it is important to mention that in human medicine, GLS of the LV is calculated as an average of the values obtained from multiple views (apical four-, apical three- and apical two-chamber view). Hertz et al. showed that this average GLS in Doberman Pinschers appeared to be less variable and therefore should be preferred over GLS obtained from a single four-chamber view whenever possible [34]. However, because the two- and three-chamber apical views are not part of the standard echocardiographic examinations in dogs, and because of the difficulties in acquiring high-quality video loops of the dogs in this study, the average GLS was not obtained with this method.

## 5. Conclusions

This study provides breed-specific RIs for conventional 2-D, M-mode, Doppler, and TDI-derived echocardiographic parameters for Dutch Sheepdogs. Furthermore, advanced 2-D STE-derived global and segmental systolic strain and global systolic SR values are reported. Selected parameters, commonly used to assess cardiac dimensions and function in dogs with PDA and MMVD, appeared to be mostly comparable to those previously reported in dogs of various breeds. However, since a few individuals might show differences potentially leading to misclassification, the complementary use of these breed-specific RIs should be considered.

BW was identified as the most important independent variable influencing most conventional echocardiographic measurements of cardiac dimensions, aligning with findings from multiple studies in both single-breed and general dog populations. HR also had notable effects on radial and circumferential strain analysis.

Inter- and intra-observer variability, as measured by CVs, was acceptable for clinical use for nearly all relevant conventional echocardiographic measurements, as well as for global strain and SR values. However, the segmental strain parameters exhibited a higher degree of variation, warranting caution in interpreting such values.

Panting and tachycardia, related to anxious behavior, were commonly observed in Dutch Sheepdogs, and should be taken into account when performing and interpreting echocardiography in dogs of this breed.

## Figures and Tables

**Table 1:** Summary statistics with 95% reference intervals of echocardiographic M-mode and B-mode parameters in 60 healthy Dutch Sheepdogs.

Variable M- and B-Mode	N/Total	Median	Mean	Standard Deviation	IQR	Min–Max	Reference Interval	*p*
IVSd (mm)	60/60	8.0	8.1	1.3	1.7	5.7–11.7	5.7–11.6	0.20
LVIDd (mm)	60/60	33.6	33.9	3.9	5.8	26.0–43.1	26.6–42.1	0.75
LVPWd (mm)	60/60	7.9	7.8	1.0	1.6	5.5–10.0	5.6–9.8	0.78
IVSs (mm)	60/60	11.3	11.4	1.5	2.3	8.4–15.4	8.7–15.0	0.36
LVIDs (mm)	60/60	23.2	22.8	4.4	5.6	13.0–32.1	13.3–31.5	0.70
LVPWs (mm)	60/60	12.5	12.6	1.7	2.2	8.4–16.8	8.8–16.4	0.95
LVIDdN	60/60	1.49	1.49	0.16	0.21	1.20–1.82	1.21–1.79	0.76
LVIDsN	60/60	0.96	0.94	0.16	0.22	0.58–1.34	0.58–1.28	0.21
TAPSE (mm)	53/60	13.1	13.6	2.7	3.3	8.4–19.7	8.5–19.6	0.19
EPSS (mm) *	58/60	2.4		1.8	2.5	0.60–8.10		<0.01
FS (%) *	60/60	32.0		7.9	10.7	20.1–54.9		0.01
HR (bpm) *	60/60	129		35	38	71–240		<0.01
LA (mm)	60/60	24.9	24.7	2.8	3.8	20.0–31.9	20.3–31.5	0.08
Ao (mm)	60/60	20.0	20.2	2.2	3.1	17.0–25.3	17.0–25.2	0.13
LA/Ao	60/60	1.21	1.22	0.11	0.17	1.00–1.46	1.01–1.45	0.56
LAD (mm)	55/60	30.7	31.1	3.3	5.0	22.8–37.3	23.3–37.2	0.36
EDV PLAX (mL) *	57/60	37.5		10.7	16.5	21.2–67.2		0.02
ESV PLAX (mL) *	57/60	14.0		6.4	9.1	5.2–34.3		0.01
EF PLAX (%)	57/60	63.2	62.6	8.2	12.8	46.8–81.0	47.3–79.7	0.67
EDV PLAX/kg	57/60	2.22	2.28	0.40	0.49	1.46–3.19	1.49–3.18	0.07
ESV PLAX/kg	57/60	0.83	0.87	0.30	0.39	0.36–1.58	0.36–1.56	0.16
EDV A4C (mL)	52/60	37.0	37.3	10.0	17.1	21.5–63.8	21.5–60.9	0.13
ESV A4C (mL)	52/60	14.7	14.9	5.6	8.2	6.3–28.1	6.5–27.0	0.06
EF A4C (%)	52/60	61.2	61.2	6.7	9.9	46.2–74.8	46.4–74.8	0.70
EDV A4C/kg	52/60	2.22	2.24	0.37	0.52	1.48–2.96	1.50–3.05	0.09
ESV A4C/kg *	52/60	0.89		0.26	0.36	0.48–1.47		0.02

Abbreviations: N/total: number of obtained measurements/total number of animals included in study; IVSd: interventricular septum thickness at end-diastole; LVIDd: left ventricular internal dimension at end-diastole; LVPWd: left ventricular posterior wall thickness at end-diastole; IVSs: interventricular septum thickness at end-systole; LVIDs: left ventricular internal dimension at end-systole; LVPWs: left ventricular posterior wall thickness at end-systole; LVIDdN: left ventricular internal luminal dimension at end-diastole normalized for BW according to calculations by Cornell et al. [3] (exponent 0.294); LVIDsN: left ventricular internal luminal dimension at end-systole normalized for BW according to calculations by Cornell et al. [3] (exponent 0.315); TAPSE: tricuspid annular plane systolic excursion; EPSS: E-point to septal separation; FS: fractional shortening; HR: heart rate; bpm: beats per minute; LA: left atrium dimension at right parasternal short-axis view at the heart base; Ao: aortic root dimension of the right parasternal right-axis view at the heart base; LA/Ao: short axis left atrial to aorta ratio; LAD: maximal left atrial anteroposterior diameter at right parasternal long-axis view; EDV: end diastolic volume; ESV: end systolic volume; EF: ejection fraction; PLAX: right parasternal long-axis four-chamber view; A4C: left apical four-chamber view; mL: milliliters N: number of obtained measurements; IQR: interquartile range; *p*: *p*-value for Shapiro–Wilk test; * normal distribution was not present (*p* < 0.05)/or small sample size (*n* < 40). For 90% confidence interval of the limits, the reader is referred to the Appendix A Table A1.

**Table 2:** Summary statistics with 95% reference intervals of conventional pulsed wave (PW), continuous wave (CW) Doppler and tissue Doppler imaging (TDI) measurements in 60 healthy Dutch Sheepdogs.

Variable Doppler/TDI	N/total	Median	Mean	Standard Deviation	IQR	Min–Max	Reference Interval	*p*
PV (m/s)	58/60	0.94	0.96	0.20	0.26	0.58–1.47	0.59–1.41	0.36
AV (m/s)	54/60	1.16	1.18	0.19	0.27	0.84–1.72	0.85–1.68	0.13
Ao (m/s) CW	60/60	1.60	1.60	0.25	0.30	1.07–2.39	1.10–2.33	0.11
MV E (m/s)	53/60	0.82	0.82	0.12	0.14	0.50–1.11	0.54–1.09	0.21
MV A (m/s)	53/60	0.59	0.62	0.16	0.24	0.32–0.98	0.33–0.97	0.45
IVRT (msec)	49/60	61.4	62.0	9.7	14.6	43.3–87.7	43.7–85.7	0.55
TV E (m/s) **	29/60	0.53		0.12	0.15	0.35–0.88		0.53
TV A (m/s) */**	29/60	0.50		0.15	0.20	0.30–0.87		0.03
TDI LVPW E’ (m/s) *	50/60	11.6		3.2	3.8	6.3–22.5		0.02
TDI LVPW A’(m/s) *	50/60	9.6		2.9	4.0	4.4–21.4		<0.01
TDI LVPW S’ (m/s) *	50/60	12.8		3.5	4.3	8.7–25.6		<0.01
TDI IVS E’ (m/s) *	47/60	8.8		2.4	2.0	4.8–15.3		0.01
TDI IVS A’ (m/s)	47/60	7.9	8.4	2.3	3.0	4.2–15.8	4.2–15.3	0.24
TDI IVS S’ (m/s)	47/60	11.3	11.9	3.0	4.6	7.0–19.6	7.0–19.4	0.19
TDI RVPW E’ (m/s)	47/60	10.9	11.9	4.0	5.4	4.7–23.1	5.1–22.4	0.12
TDI RVPW A’ (m/s)	47/60	12.2	12.5	3.9	5.4	6.1–24.0	6.2–23.1	0.19
TDI RVPW S’ (m/s) *	47/60	15.7		6.4	8.1	7.4–34.5		0.01

Abbreviations: N/total: number of obtained measurements/total number of animals included in study; PW: pulsed wave; CW: continuous wave; PV: maximal pulmonic flow velocity (PW); AV: maximal aortic flow velocity (PW); Ao: maximal aortic flow velocity (CW); MV E: peak velocity of early transmitral flow; MV A: peak velocity of late transmitral flow; IVRT: isovolumic relaxation time; TV E: peak velocity of early trans-tricuspid flow; TV A: peak velocity of late trans-tricuspid flow; TDI: tissue Doppler imaging; E’: early diastolic myocardial velocity; A’: late diastolic myocardial velocity; S’: systolic myocardial velocity; IVS: interventricular septum; LVPW: left ventricular posterior wall; RVFW: right ventricular free wall N: number of obtained measurements; IQR: interquartile range; *p*: *p*-value for Shapiro–Wilk test; * normal distribution was not present; ** small sample size (*n* < 40). For 90% confidence interval of the limits, the reader is referred to the Appendix A Table A2.

**Table 3:** Summary statistics of parameters of global strain and strain rate analyses measurements in 60 healthy Dutch Sheepdogs using vendor- independent TomTec 2-D cardiac performance analysis software 2.51 (TomTec’s CardioArena^TM^ 2022).

Variable Strain Analysis	N/Total	Median	Mean	Standard Deviation	IQR	*p*
PLAX GLS	50/60	−24.8	−26.0	5.2	6.4	0.18
PLAX GLSr *	50/60	−2.8		1.0	1.2	<0.01
SAX GCS *	53/60	−28.6		7.0	8.4	<0.01
SAX GCSr *	53/60	−3.0		1.3	2.0	<0.01
SAX GRS *	47/60	36.4		11.0	11.4	<0.01
SAX GRSr *	47/60	2.6		0.9	1.1	<0.01
A4C GLS	41/60	−19.9	−20.8	3.3	4.3	0.07
A4C GLSr	41/60	−2.0	−2.0	0.6	0.8	0.80
RV GLS	43/60	−23.4	−24.5	5.1	6.0	0.11
RV GLSr *	43/60	−2.3		1.1	1.3	<0.01

Abbreviations: N/total: number of obtained measurements/total number of animals included in study; GLS: global longitudinal strain; GLSr: global longitudinal strain rate; GRS: global radial strain; GRSr: global radial strain rate; GCS: global circumferential strain; GCSr: global circumferential strain rate; PLAX: right parasternal long-axis four-chamber view; SAX: right parasternal short-axis view at the level of the papillary muscle; A4C: left apical four-chamber view; IQR: interquartile range; *p*: *p*-value for Shapiro–Wilk test; * normal distribution was not present. For summary statistics for parameters of segmental strain and SR, the reader is referred to the Appendix A Table A3.

**Table 4:** Simple linear regression analysis of significant echocardiographic variables in healthy Dutch Sheepdogs for BW, HR and AGE (*p* < 0.05, corrected with Bonferroni correction for 43 conventional measurements *p* < 0.0012, and for 40 2-D STE measurements *p* < 0.00125).

Dependent Variable	Independent Variable	Slope	Intercept	R^2^	*p*-Value
LVIDd	BW	0.600	23.8	0.293	< 0.001
IVSd	BW	0.201	4.7	0.298	<0.001
IVSs	BW	0.213	7.8	0.241	<0.001
LVPWd	BW	0.146	5.4	0.254	<0.001
LA	BW	0.601	14.4	0.267	<0.001
EDV A4C	BW	2.181	0.982	0.591	<0.001
EDV PLAX	BW	2.289	−0.384	0.573	<0.001
ESV A4C	BW	1.047	−2.567	0.432	<0.001
ESV PLAX	BW	1.072	−3.251	0.373	<0.001
LVIDd	HR	−0.052	40.986	0.215	<0.001
LVIDs	HR	−0.076	32.650	0.292	<0.001
FS	HR	0.135	14.797	0.354	<0.001
PW PV	HR	0.003	0.529	0.313	<0.001
CW Ao	HR	0.003	1.198	0.170	0.001
PW MV E	HR	0.002	0.550	0.193	<0.001
TDI S’ IVS	HR	0.046	6.239	0.231	<0.001
TDI S’ RV	HR	0.111	3.180	0.228	<0.001
TDI E’ RV	HR	0.073	2.293	0.229	<0.001
GCS	HR	−0.092	−17.469	0.186	0.001
Circ. Midinferior sep.	HR	−0.133	−11.070	0.251	<0.001
GCSr	HR	−0.019	−0.789	0.231	<0.001
GRSr	HR	0.013	1.134	0.228	<0.001

Abbreviations: LVIDd: left ventricular internal dimension at end-diastole (M-mode); LVIDs: left ventricular internal dimension at end-systole (M-mode); IVSd: interventricular septum thickness at end-diastole (M-mode); IVSs: interventricular septum thickness at end-systole (M-mode); LVPWd: left ventricular posterior wall thickness at end-diastole (M-mode); FS: fractional shortening (M-mode); LA: left atrium dimension at right parasternal short-axis view at the heart base (B-mode); EDV: end diastolic volume (B-mode); ESV: end systolic volume (B-mode); PLAX: right parasternal long-axis four-chamber view; A4C: left apical four-chamber view; PW: pulsed wave Doppler; CW: continuous wave Doppler; PV: pulmonic valve; Ao: aorta; MV E: peak velocity of early transmitral flow; TDI: tissue Doppler imaging; E’: early diastolic myocardial velocity; S’: systolic myocardial velocity; IVS: interventricular septum; GCS: global circumferential strain; Circ.: circumferential strain with corresponding segment; GCSr: global circumferential strain rate; GRSr: global radial strain rate; BW: body weight in kilograms; HR: heart rate.

## Data Availability

Datasets are available on request from the corresponding author.

## Appendix A

**Table A1:** Summary statistics with 95% reference intervals with a 90% confidence interval for the lower and upper limits of M- and B-mode measurements in 60 healthy Dutch Sheepdogs.

Variable	N/Total	Median	Mean	Standard Deviation	IQR	Min–Max	Reference Interval	Lower 90% Confidence Interval	Upper 90% Confidence Interval	*p*
**M-mode**										
IVSd (mm)	60/60	8.0	8.1	1.3	1.7	5.7–11.7	5.7–11.6	5.6–5.9	10.1–11.7	0.20
LVIDd (mm)	60/60	33.6	33.9	3.9	5.8	26.0–43.1	26.6–42.1	26.0–27.9	40.5–43.1	0.75
LVPWd (mm)	60/60	7.9	7.8	1.0	1.6	5.5–10.0	5.6–9.8	5.5–6.0	9.3–10.0	0.78
IVSs (mm)	60/60	11.3	11.4	1.5	2.3	8.4–15.4	8.7–15.0	8.4–9.1	13.8–15.5	0.36
LVIDs (mm)	60/60	23.2	22.8	4.4	5.6	13.0–32.1	13.3–31.5	13.0–15.0	29.3–31.9	0.70
LVPWs (mm)	60/60	12.5	12.6	1.7	2.2	8.4–16.8	8.8–16.4	8.4–9.5	15.4–16.8	0.95
LVIDdN	60/60	1.49	1.49	0.16	0.21	1.20–1.82	1.21–1.79	1.20–1.24	1.72–1.82	0.76
LVIDsN	60/60	0.96	0.94	0.16	0.22	0.58–1.34	0.58–1.28	0.58–0.63	1.14–1.34	0.21
TAPSE (mm)	53/60	13.1	13.6	2.7	3.3	8.4–19.7	8.5–19.6	8.4–10.0	18.8–19.7	0.19
EPSS (mm) *	58/60	2.4		1.8	2.5	0.60–8.10				<0.01
FS (%) *	60/60	32.0		7.9	10.7	20.1–54.9				0.01
HR (bpm) *	60/60	129		35	38	71–240				<0.01
**B-Mode**										
LA (mm)	60/60	24.9	24.7	2.8	3.8	20.0–31.9	20.3–31.5	20.0–21.0	30.1–31.9	0.08
Ao (mm)	60/60	20.0	20.2	2.2	3.1	17.0–25.3	17.0–25.2	17.0–17.5	24.6–25.3	0.13
LA/Ao	60/60	1.21	1.22	0.11	0.17	1.00–1.46	1.01–1.45	1.00–1.07	1.39–1.46	0.56
LAD (mm)	55/60	30.7	31.1	3.3	5.0	22.8–37.3	23.3–37.2	22.8–27.0	36.0–37.3	0.36
EDV PLAX (mL) *	57/60	37.5		10.7	16.5					0.02
ESV PLAX (mL) *	57/60	14.0		6.4	9.1					0.01
EF PLAX (%)	57/60	63.2	62.6	8.2	12.8	46.8–81.0	47.3–79.7	46.8 -50.3	75.3–81.0	0.67
EDV PLAX/kg	57/60	2.22	2.28	0.40	0.49	1.46–3.19	1.49–3.18	1.46–1.71	3.05–3.19	0.07
ESV PLAX/kg	57/60	0.83	0.87	0.30	0.39	0.36–1.58	0.36–1.56	0.36–0.44	1.39–1.58	0.16
ESV A4C (mL)	52/60	14.7	14.9	5.6	8.2	6.3–28.1	6.5–27.0	6.3–7.4	24.1–28.1	0.06
EF A4C (%)	52/60	61.2	61.2	6.7	9.9	46.2–74.8	46.4–74.7	46.2–50.7	71.7–74.8	0.70
EDV A4C/kg	52/60	2.22	2.24	0.37	0.52	1.48–2.96	1.50–3.05	1.48–1.80	2.95–3.06	0.09
ESV A4C/kg *	52/60	0.89		0.26	0.36	0.48–1.47				0.02

Abbreviations: N/total: number of obtained measurements/total number of animals included in study; IVSd: interventricular septum thickness at end-diastole; LVIDd: left ventricular internal dimension at end-diastole; LVPWd: left ventricular posterior wall thickness at end-diastole; IVSs: interventricular septum thickness at end-systole; LVIDs: left ventricular internal dimension at end-systole; LVPWs: left ventricular posterior wall thickness at end-systole; LVIDdN: left ventricular internal luminal dimension at end-diastole normalized for body weight according to calculations by Cornell et al. [3] (exponent 0.294); LVIDsN: left ventricular internal luminal dimension at end-systole normalized for body weight according to calculations by Cornell et al. [3] (exponent 0.315); TAPSE: tricuspid annular plane systolic excursion; EPSS: E-point to septal separation; FS: fractional shortening; HR: heart rate; bpm: beats per minute; LA: left atrium dimension at right parasternal short-axis view at the heart base; Ao: aortic root dimension of the right parasternal right-axis view at the heart base; LA/Ao: short axis left atrial to aorta ratio; LAD: maximal left atrial anteroposterior diameter at right parasternal long-axis view; EDV: end diastolic volume; ESV: end systolic volume; EF: ejection fraction; PLAX: right parasternal long-axis four-chamber view; A4C: left apical four-chamber view; *p*: *p*-value for Shapiro–Wilk test * no normal distribution was present/or small sample size.

**Table A2:** Summary statistics of echocardiographic Doppler and TDI measurements with 95% reference intervals with a 90% confidence interval for the lower and upper limits in 60 healthy Dutch Sheepdogs.

Variable	N/Total	Median	Mean	Standard Deviation	IQR	Min–Max	Reference Interval	Lower 90% Confidence Interval	Upper 90% Confidence Interval	*p*
**Doppler**										
PV (m/s) PW	58/60	0.94	0.96	0.20	0.26	0.58–1.47	0.59–1.41	0.58–0.66	1.33–1.47	0.36
AV (m/s) PW	54/60	1.16	1.18	0.19	0.27	0.84–1.72	0.85–1.68	0.84–0.92	1.47–1.72	0.13
Ao (m/s) CW	60/60	1.60	1.60	0.25	0.30	1.07–2.39	1.10–2.33	1.07–1.19	1.97–2.39	0.11
MV E (m/s)	53/60	0.82	0.82	0.12	0.14	0.50–1.11	0.54–1.09	0.50–0.65	1.06–1.11	0.21
MV A (m/s)	53/60	0.59	0.62	0.16	0.24	0.32–0.98	0.33–0.97	0.32–0.38	0.89–0.98	0.45
IVRT (msec)	49/60	61.4	62.0	9.7	14.6	43.3–87.7	43.7–85.7	43.3–46.9	74.0–87.7	0.55
TV E (m/s) **	29/60	0.53		0.12	0.15	0.35–0.88				0.53
TV A (m/s) */**	29/60	0.50		0.15	0.20	0.30–0.87				0.03
**TDI**										
TDI LVPW E’ (m/s) *	50/60	11.6		3.2	3.8	6.3–22.5				0.02
TDI LVPW A’(m/s) *	50/60	9.6		2.9	4.0	4.4–21.4				<0.01
TDI LVPW S’ (m/s) *	50/60	12.8		3.5	4.3	8.7–25.6				<0.01
TDI IVS E’ (m/s) *	47/60	8.8		2.4	2.0	4.8–15.3				0.01
TDI IVS A’ (m/s)	47/60	7.9	8.4	2.3	3.0	4.2–15.8	4.2–15.3	4.2–5.0	11.8–15.8	0.24
TDI IVS S’ (m/s)	47/60	11.3	11.9	3.0	4.6	7.0–19.6	7.0–19.4	7.0–8.4	17.7–19.6	0.19
TDI RVPW E’ (m/s)	47/60	10.9	11.9	4.0	5.4	4.7–23.1	5.1–22.4	4.7–6.9	17.9–23.1	0.12
TDI RVPW A’ (m/s)	47/60	12.2	12.5	3.9	5.4	6.1–24.0	6.2–23.1	6.1–6.9	18.7–24.0	0.19
TDI RVPW S’ (m/s) *	47/60	15.7		6.4	8.1	7.4–34.5				0.01

Abbreviations: N/total: number of obtained measurements/total number of animals included in study; PW: pulsed wave; CW: continuous wave; PV: maximal pulmonic flow velocity (PW); AV: maximal aortic flow velocity (PW); Ao: maximal aortic flow velocity (CW); MV E: peak velocity of early transmitral flow; MV A: peak velocity of late transmitral flow; IVRT: isovolumic relaxation time; TV E: peak velocity of early trans-tricuspid flow; TV A: peak velocity of late trans-tricuspid flow; TDI: tissue Doppler imaging; E’: early diastolic myocardial velocity; A’: late diastolic myocardial velocity; S’: systolic myocardial velocity; IVS: interventricular septum; LVPW: left ventricular posterior wall; RVFW: right ventricular free wall; N: number of obtained measurements; IQR: interquartile range; *p*: *p*-value for Shapiro–Wilk test; * normal distribution was not present; ** small sample size (*n* < 40).

**Table A3:** Summary statistics of parameters of strain analysis (global, segmental, and strain rate) in 60 healthy Dutch Sheepdogs.

Variable Strain Analysis	N/Total	Median	Mean	Standard Deviation	IQR	Min–Max	*p*
PLAX GLS	50/60	−24.8	−26.0	5.2	6.4	−40.9; −16.4	0.18
PLAX FW basal	50/60	−24.2	−23.8	6.2	7.6	−40.2; −10.9	0.65
PLAX FW mid	50/60	−24.5	−25.0	6.4	7.5	−43.4; −11.5	0.42
PLAX FW apical	50/60	−30.3	−31.7	8.5	11.1	−55.8; −14.0	0.41
PLAX IVS apical	50/60	−33.6	−35.2	9.5	16.1	−56.8; −19.6	0.14
PLAX IVS mid	50/60	−23.9	−23.9	5.7	6.9	−41.6; −6.7	0.33
PLAX IVS basal	50/60	−20.7	−20.8	6.4	8.8	−39.8; −8.4	0.79
PLAX GLSr *	50/60	−2.8	−2.9	1.0	1.2	−5.8; −1.7	<0.01
GCS *	53/60	−28.6	−29.9	7.0	8.4	−50.8; −17.6	<0.01
Circ mid anterior	53/60	−29.2	−29.5	8.3	10.9	−53.4; −7.6	0.81
Circ mid anterior lateral	53/60	−35.5	−36.0	8.2	10.0	−54.6; −15.6	0.78
Circ mid inferior lateral	53/60	−29.1	−30.1	9.5	14.8	−54.6; −10.6	0.76
Circ mid inferior	53/60	−29.4	−30.8	8.9	10.8	−54.4; −14.4	0.20
Circ mid inf. Septum *	53/60	−27.0	−29.0	8.7	10.1	−54.6; −14.7	<0.01
Circ mid ant. Septum *	53/60	−25.7	−28.1	8.8	12.3	−49.8; −13.6	0.01
GCSr *	53/60	−3.0	−3.4	1.3	2.0	−7.1; −1.5	<0.01
GRS *	47/60	36.4	37.9	11.0	11.4	20.3; 75	<0.01
Radial mid anterior	47/60	37.7	39.3	15.0	21.0	10.7; 71.3	0.20
Radial mid anterior lat.	47/60	37.5	37.6	13.8	16.0	6.8; 86.5	0.09
Radial mid inferior lat. *	47/60	42.3	44.1	16.4	22.5	19.5; 108.8	<0.01
Radial mid inferior *	47/60	40.9	43.0	13.9	16.8	20.5; 101.7	<0.01
Radial mid inf. septum	47/60	35.1	36.3	12.5	16.1	16.3; 68.0	0.07
Radial mid ant. Septum	47/60	30.1	32.5	12.0	14.2	9.8; 63.4	0.16
GRSr *	47/60	2.6	2.8	0.9	1.1	1.6; 5.4	<0.01
A4C GLS	41/60	−19.9	−20.8	3.3	4.3	−30.1; −14.9	0.07
A4C IVS basal	40/60	−18.3	−18.0	3.8	4.7	−25.0; −9.8	0.75
A4C IVS mid	41/60	−20.9	−20.4	4.6	5.9	−29.8; −9.2	0.85
A4C IVS apical *	41/60	−24.8	−25.4	6.2	7.8	−46.0; −11.4	0.03
A4C FW apical	41/60	−22.7	−24.0	7.0	12.2	−38.4; −8.3	0.37
A4C FW mid	41/60	−23.6	−23.2	5.6	9.0	−34; −12.2	0.64
A4C FW basal	41/60	−21.5	−21.3	4.5	6.6	−30.9; −13.9	0.36
A4C GLSr	41/60	−2.0	−2.0	0.6	0.8	−3.4; −0.9	0.80
RV GS	43/60	−23.4	−24.5	5.1	6.0	−37.6; −16.7	0.11
RV FW base	43/60	−30.1	−29.7	6.6	10.8	−44.1; −14.4	0.83
RV FW mid	43/60	−34.9	−33.7	7.5	8.7	−53.5; −19.0	0.81
RV FW apical	43/60	−24.4	−26.5	8.9	9.9	−48.6; −10.7	0.06
RV IVS apical	41/60	−18.8	−19.6	7.3	11.0	−34.1; −5.9	0.71
RV IVS mid	43/60	−23.1	−23.5	5.3	7.1	−37.7; −14.5	0.65
RV IVS base	43/60	−21.2	−22.7	6.9	8.5	−40.8; −9.1	0.12
RV GSr *	43/60	−2.3	−2.5	1.1	1.3	−6.6; −0.9	<0.01

Abbreviations: N/total: number of obtained measurements/total number of animals included in study; PLAX: right parasternal long-axis four-chamber view; A4C: left apical four-chamber view; FW: free wall; IVS: inter ventricular septum; RV: right ventricle; GLS: global longitudinal strain; GLSr: global longitudinal strain rate; GRS: global radial strain; GRSr: global radial strain rate; GCS: global circumferential strain; GCSr: global circumferential strain rate; RV GS: right ventricular global strain; RV GSr: right ventricular global strain rate; Circ.: circumferential strain with the corresponding segment; Radial: radial strain with the corresponding segment; lat.: lateral; IQR: interquartile range; *p*: *p*-value for Shapiro–Wilk test; * normal distribution was not present.

## Appendix B

**Table A4:** Intra-observer (within-day and between-day) and inter-observer variability (CV in %) of conventional echocardiographic M-mode, B-mode, PW/CW Doppler and TDI measurements obtained from PLAX, SAX and A4C views in six dogs.

Variable	Intra-Observer (Within-Day) CV (%)	Intra-Observer (Between-Day) CV (%)	Inter-Observer CV (%)
IVSd	3.6	2.9	7.4
LVIDd	1.1	2.4	3.5
LVPWd	2.9	3.9	6.5
IVSs	2.2	3.6	3.9
LVIDs	1.4	2.8	6.4
LVPWs	3.6	3.9	7.2
LVIDdN	1.1	2.4	4.2
LVIDsN	1.6	2.8	8.4
FS (%)	4.4	3.5	6.1
EPSS	13.3	12.3	13.3
TAPSE	2.6	2.5	7.9
LAD	1.1	1.5	1.2
Ao	1.0	1.0	1.4
LA	3.2	3.5	3.6
LA/Ao	3.8	2.9	5.6
ESV PLAX	6.1	7.5	11.5
ESV A4C	3.6	10.9	25.3
EDV PLAX	1.7	4.0	6.5
EDV A4C	3.6	9	15.9
EF PLAX (%)	4.1	3.5	5.7
EF A4C (%)	5.3	5.9	7.1
PV	1.1	1.8	9.9
AV	2.2	3.8	1.0
Ao	1.4	1.1	2.1
MV E	3.7	1.5	8.1
MV A	2.4	3.6	14.3
MV E/A	2.0	2.1	15.1
IVRT	4.5	4.6	18.0
TV E	8.3	3.7	11.8
TV A	8.9	3.5	10.9
TV E/A	11.6	4.4	14.2
S’ lateral	2.2	4.8	12.8
E’ lateral	2.3	6.9	13.6
A’ lateral	1.3	2.3	10.6
S’ IVS	2.2	6.1	13.5
E’ IVS	2.8	9.2	13.7
A’ IVS	3.3	8.9	9.1
S’ RVFW	2.2	4.3	5.3
E’ RVFW	2.2	2.4	8.9
A’ RVFW	2.3	3.0	6.6

Abbreviations: CV: coefficient of variation; PLAX: right parasternal long-axis four-chamber view; SAX: right parasternal short-axis view; A4C: left apical four-chamber view; IVSd: interventricular septum thickness at end-diastole; LVIDd: left ventricular internal dimension at end-diastole; LVPWd: left ventricular posterior wall thickness at end-diastole; IVSs: interventricular septum thickness at end-systole; LVIDs: left ventricular internal dimension at end-systole; LVPWs: left ventricular posterior wall thickness at end-systole; LVIDdN: left ventricular internal luminal dimension at end-diastole normalized for bodyweight according to calculations by Cornell et al. [3]; LVIDsN: left ventricular internal luminal dimension at end-systole normalized for bodyweight according to calculations by Cornell et al. [3]; FS: fractional shortening; EPSS: E-point to septal separation; TAPSE: tricuspid annular plane systolic excursion; LAD: maximal left atrial anteroposterior diameter at right parasternal long-axis view; Ao: aortic root dimension of the right parasternal right-axis view at the heart base; LA: left atrium dimension at right parasternal short-axis view at the heart base; LA/Ao: short-axis left atrial to aorta ratio; ESV: end systolic volume; EDV: end diastolic volume; EF: ejection fraction; PLAX: right parasternal long-axis four-chamber view; A4C: left apical four-chamber view; PW: pulsed wave; CW: continuous wave; PV: maximal pulmonic flow velocity (PW); AV: maximal aortic flow velocity (PW); Ao: maximal aortic flow velocity (CW); MV E: peak velocity of early transmitral flow; MV A: peak velocity of late transmitral flow; IVRT: isovolumic relaxation time; TV E: peak velocity of early trans-tricuspid flow; TV A: peak velocity of late trans-tricuspid flow; TDI: tissue Doppler imaging; S’: systolic myocardial velocity; E’: early diastolic myocardial velocity; A’: late diastolic myocardial velocity; IVS: interventricular septum; LVPW: left ventricular posterior wall; RVFW: right ventricular free wall.

**Table A5:** Intra-observer (within-day and between-day) and inter-observer variability (CV in %) of STE-derived systolic strain (global and segmental) and strain rate from PLAX, SAX and A4C views in six dogs.

Variable	Intra-Observer (Within-Day) CV (%)	Intra-Observer (Between-Day) CV (%)	Inter-Observer CV (%)
PLAX GLS	5.2	10.7	7.2
PLAX FW basal	15.3	11.2	11.3
PLAX FW mid	13.9	15.4	12.9
PLAX FW apical	18.4	14.0	12.3
PLAX IVS apical	19.8	23.2	12.4
PLAX IVS mid	4.8	9.1	11.4
PLAX IVS basal	11.3	8.9	11.3
PLAX GLSr	6.1	5.3	5.0
GCS	4.6	5.5	5.5
Circumferential mid anterior	5.7	7.2	5.9
Circumferential mid anterior lateral	6.5	6.6	12.8
Circumferential mid inferior lateral	8.1	18.1	7.9
Circumferential mid inferior	8.0	7.1	4.8
Circumferential mid inferior septal	10.6	8.5	7.1
Circumferential mid anterior septal	12.1	9.9	14.8
GCSr	4.6	9.1	3.3
GRS	14.5	14.0	7.9
Radial mid anterior	37.1	33.8	17.1
Radial mid anterior lateral	32.8	16.2	14.9
Radial mid inferior lateral	17.2	11.1	11.4
Radial mid inferior	14.3	11.8	6.1
Radial mid inferior septal	10.3	16.5	11.9
Radial mid anterior septal	16.5	32.7	18.8
GRSr	5.6	9.5	4.2
A4C GLS	7.1	8.3	11.0
A4C IVS basal	10.2	17.8	9.8
A4C IVS mid	18.2	25.4	11.5
A4C IVS apical	21.4	13.5	21.4
A4C FW apical	16.6	10.8	12.9
A4C FW mid	11.0	14.0	11.1
A4C FW basal	10.0	11.6	9.6
A4C GLSr	9.1	6.1	8.1
RV GS	6.7	12.9	13.1
RV FW basal	15.9	30.2	24.8
RV FW med	6.7	16.7	17.4
RV FW apical	15.5	24.3	25.9
RV septum apical	22.5	25.0	10.2
RV septum med	14.5	12.4	11.3
RV septum basal	13.4	11.5	16.0
RV GSr	10.1	16.4	12.0

Abbreviations: CV: coefficient of variation; PLAX: right parasternal long-axis four-chamber view; SAX: right parasternal short-axis view; A4C: left apical four-chamber view; FW: free wall; IVS: inter ventricular septum; RV: right ventricle; GLS: global longitudinal strain; GLSr: global longitudinal strain rate; GCS: global circumferential strain; GCSr: global circumferential strain rate; GRS: global radial strain; GRSr: global radial strain rate; RV GS: right ventricular global strain; RV GSr: right ventricular global strain rate.
